# Pulmonary Hemodynamics and Six-Minute Walk Test Outcomes in Patients with Interstitial Lung Disease

**DOI:** 10.1155/2016/3837182

**Published:** 2016-05-18

**Authors:** Osamu Nishiyama, Ryo Yamazaki, Hiroyuki Sano, Takashi Iwanaga, Yuji Higashimoto, Hiroaki Kume, Yuji Tohda

**Affiliations:** Department of Respiratory Medicine and Allergology, Kindai University Faculty of Medicine, 377-2 Onohigashi, Osakasayama, Osaka 589-8511, Japan

## Abstract

*Background*. Six-minute walk test (6MWT) has 3 measurement outcomes, which are walk distance, desaturation, and symptom. The aim of this study was to examine whether routinely measured right-heart catheter (RHC) data correlate with 6MWT outcomes in patients with interstitial lung disease (ILD).* Methods*. Between June 2010 and December 2012, consecutive patients with ILD who underwent evaluation, including pulmonary function test, hemodynamic studies with right-heart catheter, and 6MWT as routine general practice, were recruited. Correlates of 3 outcomes of 6MWT were examined to reveal significant predictors.* Results*. Forty-six patients consisting of 20 with idiopathic pulmonary fibrosis, 14 with collagen vascular disease associated ILD, and 12 with other idiopathic interstitial pneumonia were recruited (mean % predicted FVC: 76.7 ± 17.1%). Several physiological variables, including mean pulmonary artery pressure (PAP) and pulmonary vascular resistance (PVR), were correlated with each 6MWT outcome. Stepwise multivariate regression analyses showed that % predicted FVC and % predicted DLco were independent predictors of walk distance (*r* ^2^ = 0.35, *p* = 0.0002). For SpO_2_ at the end of 6MWT, % predicted DLco and PVR were selected as independent predictors (*r* ^2^ = 0.46, *p* < 0.0001). For dyspnea at the end of 6MWT, % predicted DLco was only one predictor (*r* ^2^ = 0.18, *p* = 0.005).* Conclusion*. Mean PAP had little impact on 6MWT outcomes in ILD patients who were nonselectively recruited, although PVR was one of predictors of desaturation.

## 1. Introduction

The 6-min walk test (6MWT) is the most frequently used exercise capacity assessment for patients with chronic lung disease. Exercise tolerance assessed with 6MWT is submaximal capacity because most patients do not achieve maximal effort during the test [1]. The test itself requires an unobstructed walking path but no exercise equipment and little advanced training to administer. It evaluates the integrated global response of the pulmonary, cardiovascular, and neuromuscular systems. Therefore, it is considered difficult to provide specific information regarding which system contributes to exercise limitation, something a cardiopulmonary exercise test would provide [2, 3].

However, correlates of a 6-min walk distance have been studied in several diseases. In the field of lung disease, chronic obstructive pulmonary disease (COPD) has been earnestly investigated. Several factors which predict a reduced 6-min walk distance in patients with COPD were previously found. Not only decreased forced expiratory flow in one second (FEV_1_) [1], but also older age [4], female sex [5], and higher body mass index [5, 6] were reportedly associated with reduced walk distance. In patients with interstitial lung disease (ILD), however, significant predictors of 6MWT outcomes have not been sufficiently investigated.

When performing 6MWT, not only distance walked but also O_2_ saturation and dyspnea rating are usually recorded. In patients with idiopathic pulmonary fibrosis (IPF), for example, both walk distance and desaturation at 6MWT are significant prognostic markers [7–11]. Thus, evaluating 6MWT outcomes is important in ILD as well. Moreover, desaturation during 6MWT is more severe for IPF than those with COPD, even though resting oxygenation between the two is equivalent [12]. This might be a consequence of the differing pathophysiology between restrictive and obstructive lung disease. Mechanisms of exercise intolerance are also different, so the correlates of 6MWT outcomes should be investigated separately in obstructive and restrictive lung disease.

Recently, a high prevalence of pulmonary hypertension was found in IPF, and evaluating pulmonary artery pressure has been becoming important [13–16]. Moreover, pulmonary hypertension in ILD is associated with exercise-induced desaturation [14]. Therefore, pulmonary hemodynamics in ILD might be involved in reduced exercise tolerance and be associated with 6MWT parameters, including walk distance, desaturation, and symptoms. However, pulmonary artery pressure is not usually measured in clinical practice unless presence of pulmonary hypertension is strongly suspected.

Consequently, the aim of this study was to examine whether routinely measured right-heart catheter (RHC) data correlate with 6MWT outcomes. Not only walk distance but also desaturation and dyspnea are included in outcomes and a comparison was made among these three.

## 2. Methods

### 2.1. Patients

Pulmonary function tests (PFTs), 6MWT, and RHC are part of the regular evaluation for patients with ILD when diagnosed at our university hospital. Inclusion criteria for this study were as follows: diagnosis of ILD; having undergone evaluation between June 2010 and December 2012; age ≧18 years; the absence of any recent (i.e., <3 months) acute worsening of ILD including infection, pulmonary embolism, and acute exacerbation of ILD; the absence of comorbid illness which might influence the evaluation of 6MWT and RHC, such as cardiovascular disease, chronic pulmonary embolism, COPD, and asthma. Patients with long-term oxygen therapy who could not remove their oxygen at the time of both 6MWT and RHC examinations were excluded. Patients with mean pulmonary artery pressure (PAP) ≧35 were also excluded because of possibility that pulmonary artery hypertension is the main pathophysiology in such patients. Since this study was based on retrospective analysis, the informed consent requirement was waived. The study protocol was approved by the ethics committee of Kindai University Faculty of Medicine (number 25-012).

### 2.2. PFTs

PFTs included spirometry and single-breath measurements of diffusing capacity (DLco) (CHESTAC-8800; Chest, Tokyo, Japan) and arterial blood gas analysis, which were all performed according to the American Thoracic Society standards [17, 18]. Results were expressed in absolute values and as a percentage of Japanese normal predicted values [19].

### 2.3. Hemodynamic Studies

RHC was performed using a Swan-Ganz catheter percutaneously via either the cervical or femoral vein. Hemodynamic variables including mean PAP, systolic and diastolic right ventricular pressure, mean right arterial pressure (RAP), pulmonary capillary wedge pressure (PCWP), and mean systemic arterial pressure were measured. Cardiac output (CO) was also measured by the thermodilution method. Pulmonary vascular resistance was calculated by dividing (mean pulmonary artery pressure − pulmonary capillary wedge pressure) by cardiac output.

### 2.4. 6MWT

6MWT was conducted in all patients who participated in the study, according to the ATS statement [1]. Briefly, all patients were tested under the standardized condition by trained physicians. The patients wore lightweight pulse oximeters (WristOx*™*, NONIN, Inc., Minneapolis, USA), and baseline heart rate and transcutaneous oxygen saturation (SpO_2_) were measured. Then, patients were instructed to walk as far as possible for 6 min. The physician who conducted the test did not walk with the patients but checked and recorded SpO_2_ and heart rates on the pulse oximeter immediately after the test. The distance the patients walked and dyspnea rating immediately after the test were also recorded. To evaluate dyspnea rating, patients were asked to rate their dyspnea using the modified Borg scale, by selecting a number from 0 to 10, with 0 being no appreciable dyspnea and 10 being maximal sustainable dyspnea [20].

### 2.5. Statistical Analyses

Pearson correlation coefficients (*r*) between 6MWT outcomes and various physiological parameters including RHC data were determined. The 6MWT outcomes included walk distance, SpO_2_ at the end of 6MWT, and dyspnea assessed with the Borg scale. The interrelationships among 6MWT outcomes were also examined. In the subsequent multivariate model, a stepwise regression analysis was performed using each 6MWT outcome as a dependent variable to select more significant variables. Any variables which had been revealed to have a significant correlation with the dependent variable were introduced in the multivariate model as potential predictors. As for pulmonary function variables such as forced vital capacity (FVC) and DLco, % predicted values were used in the multivariate model to avoid the effect of anthropometric factors. All tests were performed at a significance level of 0.05. Analyses were performed with the PASW statistical package ver.18 (SPSS Japan Inc., Tokyo, Japan), and all values are presented as mean ± standard deviation (SD) unless otherwise indicated.

## 3. Results

### 3.1. Patient Characteristics

Forty-six consecutive patients with ILD were included in the study. Anthropometry and diagnoses of the patients are shown in Table 1. Of 46 patients, 20 were diagnosed with IPF according to the official ATS/ERS/JRS/ALAT statement [21]. Fourteen were diagnosed as having collagen vascular disease associated interstitial lung disease (CVD-ILD), which included 8 patients with rheumatoid arthritis, 3 with systemic sclerosis, 2 with dermatomyositis, and 2 with Sjögren's syndrome. The other 12 patients were not diagnosed with specific ILD, and therefore they were diagnosed as simply having idiopathic interstitial pneumonia (IIP).

### 3.2. PFT, Arterial Gas Analysis, Hemodynamics, and 6MWT Outcomes

**Figure 1:**
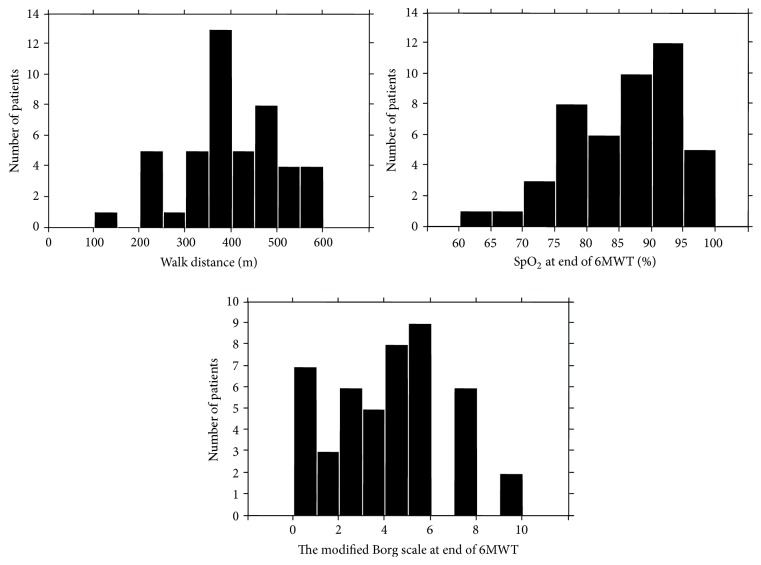
Histogram of patients for 6-min walk distance, SpO_2_ at end of 6MWT, and dyspnea assessed with modified Borg scale at the end of the test. SpO_2_ transcutaneous oxygen saturation; 6MWT: 6-min walk test.

PFT data, arterial blood analysis, hemodynamics, and results of 6MWT are shown in Table 2. The patients had a mild decrease in % predicted FVC, consistent with a mild restrictive defect. Reduction in DLco was moderate and decrease in PaO_2_ was relatively mild. RHC data show the mean PAP and mean PCWP within a normal range. However, there were 2 patients whose mean PAP was ≧25 mmHg, high enough to be diagnosed with pulmonary hypertension [22, 23]. The mean PAPs of each of these 2 patients were 26 with IIP and 27 with dermatomyositis. In 6MWT outcomes, the mean walk distance was 394 m, although the distance was widely distributed from person to person. The mean end-test SpO_2_ was 85.4%, showing mild desaturation. In fact, 23 of 46 patients (50.0%) showed desaturation of less than 88% at the end of the 6MWT. The mean dyspnea level assessed with the modified Borg scale was 3.7, although it ranged between extremes. As shown in Figure 1, actual values of each outcome were widely distributed.

### 3.3. Correlates of 6MWT Outcomes

The results of single correlation analysis for 6MWT outcomes are shown in Table 3. For walk distance, weight (*r* = 0.31, *p* = 0.04), FVC (*r* = 0.49, *p* = 0.0005), % predicted FVC (*r* = 0.39, *p* = 0.007), DLco (*r* = 0.61, *p* < 0.0001), % predicted DLco (*r* = 0.46, *p* = 0.002), PaO_2_ (*r* = 0.43, *p* = 0.002), and PVR (*r* = −0.33, *p* = 0.02) were revealed to be significant correlates.

For SpO_2_ at the end of 6MWT, DLco (*r* = 0.47, *p* = 0.001), % predicted DLco (*r* = 0.49, *p* = 0.0007), PaO_2_ (*r* = 0.43, *p* = 0.003), mean PAP (*r* = −0.50, *p* = 0.0003), and PVR (*r* = −0.63, *p* < 0.0001) were significant correlates.

For dyspnea at end of 6MWT assessed with the modified Borg scale, factors which showed significant correlation were DLco (*r* = −0.38, *p* = 0.01), % predicted DLco (*r* = −0.42, *p* = 0.004), and mean PAP (*r* = 0.32, *p* = 0.03).

The results of stepwise multivariate regression analyses which were performed to reveal more significant parameters are shown in Table 4. % predicted FVC and % predicted DLco were as independent predictors of walk distance (*r* ^2^ = 0.35, *p* = 0.0002). For SpO_2_ at the end of 6MWT, % predicted DLco and PVR were selected as independent predictors (*r* ^2^ = 0.46, *p* < 0.0001). For dyspnea at the end of 6MWT, only % predicted DLco was a significant independent predictor (*r* ^2^ = 0.18, *p* = 0.005).

## 4. Discussion

In the present study, we analyzed whether pulmonary hemodynamics significantly correlate with and predict each 6MWT outcome, including walk distance, SpO_2_, and dyspnea at the end of the test, and also examined to see if there were any differences among different 6MWT outcomes in patients with ILD who were nonselectively recruited. As results of univariate analysis, mean PAP and/or PVR as well as % predicted DLco were significantly correlated with all 3 outcomes of 6MWT. In stepwise multivariate regression analyses, pulmonary hemodynamics data was excluded from significant correlates of 6MWT outcomes except SpO_2_ at end of 6MWT. Furthermore, some differences were observed among predictors for 3 outcomes of 6MWT. As for walk distance, % predicted FVC and % predicted DLco were independent predictors. In contrast, as for SpO_2_ at the end of the test, % predicted DLco and PVR were independent predictors. As for dyspnea at the end of the test, % predicted DLco was only one predictor.

In patients with COPD, several factors which predict 6-min walk distance have been reported. It has been demonstrated that 6-min walk distance is shorter in patients with an advanced age and a higher BMI, in patients with severe airflow obstruction, and in women [1, 4–6]. However, these factors explain only a small part of the variation among individuals [24]. Some other factors which increase exertional dyspnea, such as dynamic hyperinflation, may play a role in the variability of walk distance [24].

There have been some reports examining the impact of pulmonary hemodynamics on exercise capacity in ILD [25–28]. Those reports equally demonstrated that mPAP had significant negative impact on exercise capacity. In our study, however, mPAP was not selected as predictor of any 6MWT outcomes in multivariate analysis, although it was correlated with each outcome of 6MWT in univariate analysis. It seems that this discrepancy arose because of differences in patient characteristics. Only 4% of patients had pulmonary hypertension of ≧25 mmHg in our study while more patients did in other studies. One of the reasons for this difference may be that RHC was performed as part of a routine regular evaluation for ILD in our hospital. Another reason may be that some patients with pulmonary hypertension could have been be excluded from our study, because patients with long-term oxygen therapy who could not discontinued their oxygen apparatus at the time of 6MWT and RHC examinations both were excluded from the study. The exclusion criteria for the present study of patients with mean PAP ≧35 also might contribute. For these reasons, it can be said that the results of the present study reflect characteristics of patients with mean PAP within normal range to mild pulmonary hypertension, which also may reflect the real world in clinical practice. The presence of pulmonary hypertension is important and is one of predicting factors of longevity in IPF patients [13–16]. However, in patient populations with mean PAP within normal range to mild pulmonary hypertension, pulmonary artery pressure had little influence on 6MWT outcomes in multivariate analysis, although even pulmonary artery pressure less than 25 mmHg reportedly influences the longevity of patients with IPF [15]. It can be assumed in clinical practice that the presence of pulmonary hypertension should be investigated separately from evaluations for pulmonary function and exercise capacity in ILD patients. Of course, further study is needed to confirm the importance of pulmonary artery pressure on 6MWT outcomes in each specific disease.

However, a PVR was selected as one of the predictors of desaturation at 6MWT, although it was not correlated with 6-min walk distance. Abnormality in pulmonary hemodynamic influences only desaturation after exercise in this patient population.

The present study should be considered in light of its strengths and limitations. Analysis including hemodynamic variables measured with routine RHC examination allowed us to exclude selection bias. If RHC was performed only for patients who most likely had pulmonary hypertension, the rate of patients with severe lung impairment or with a factor of pulmonary artery hypertension would have increased, and the patient population would have been slanted. On the contrary, there are some limitations which should be addressed in the present study. First, as mentioned above, it was possible that some patients with pulmonary hypertension were excluded from the study, because patients who could not take off their oxygen apparatus at the time of both examinations were excluded. If more patients with severe pulmonary hypertension ≧25 mgHg of mPAP were included in the study, other results might have been obtained. It may also be true that ILD patients with pulmonary hypertension should be investigated separately. Second, the diagnosis of diseases varied. The patient population consisted of IPF, IIP with no specific classification, and CVD-ILD. Because differences might exist among pathophysiologies of ILD, further study which enrolls patients with a specific disorder, such as IPF only, should be considered.

In conclusion, % predicted FVC and % predicted DLco were predictors of 6-min walk distance. % predicted DLco and PVR were predictors of desaturation at 6MWT. Only % predicted DLco was a predictor of dyspnea at 6MWT in patients with ILD. Consequently, mean PAP had little impact on 6MWT outcomes in ILD patients who were nonselectively recruited, although a PVR was one of predictors of desaturation.

## Figures and Tables

**Table 1:** Patient characteristics.

Variables	Values
Sex, number (%)	
Male	28 (61)
Female	18 (39)

Age, yr	68.9 ± 10.1
Height, cm	159.2 ± 8.8
Weight, kg	58.1 ± 12.6

Diagnosis, number	
IPF	20
IIP (no specific diagnosis)	12
CVD-ILD	14

The number of patients was 46. Data are presented as mean ± standard deviation (SD), unless otherwise indicated.

IPF: idiopathic pulmonary fibrosis; IIP: idiopathic interstitial pneumonia; CVD-ILD: connective tissue disease associated interstitial lung disease.

**Table 2:** Pulmonary function, arterial gas analysis, hemodynamics, and 6 min walk test outcomes.

Variables	Mean ± SD	Range
Pulmonary function		
FVC, L	2.3 ± 0.8	0.9–4.4
FVC, % predicted	76.7 ± 17.1	41.5–122.0
DLco, mL/min/mmHg	10.1 ± 3.2	4.8–17.0
DLco, % predicted	62.9 ± 16.0	37.8–118.1

Arterial gas analysis		
PaO_2_, mmHg	76.6 ± 10.6	58.0–101.7
PaCO_2_, mmHg	40.6 ± 4.4	31.1–48.8
pH	7.41 ± 0.02	7.38–7.47

Hemodynamics		
Mean AP, mmHg	91.3 ± 12.3	66.6–114.0
RAP, mmHg	2.9 ± 2.8	0.4–8.1
Mean PAP, mmHg	16.6 ± 4.8	7.0–27.0
PCWP, mmHg	6.5 ± 3.3	0–15.0
CO, L/min	4.7 ± 1.1	3.0–8.0
Cardiac index, L/min/m^2^	3.0 ± 0.6	1.8–4.6
PVR, dyne·s/cm^5^	177 ± 81	0–342

6MWT outcomes		
Walk distance, m	394 ± 102	120–580
SpO_2_ at end of the test, %	85.4 ± 8.3	62–97
Dyspnea at end of the test	3.7 ± 2.6	0–10

The number of patients was 46 except for DLco which was 44 because 2 could not perform the examination for % DLco due to low pulmonary function.

Data are presented as mean ± standard deviation (SD). Dyspnea was assessed with the modified Borg scale.

FVC: forced vital capacity; DLco: diffusing capacity of carbon monoxide; PaO_2_: partial arterial pressure of oxygen; PaCO_2_: partial arterial pressure of carbon dioxide; AP: systemic arterial pressure; RAP: right arterial pressure; PAP: pulmonary artery pressure; PCWP: pulmonary capillary wedge pressure; CO: cardiac output; PVR: pulmonary vascular resistance; SpO_2_: transcutaneous oxygen saturation; 6MWT: 6 min walk test.

**Table 3:** Pearson correlation coefficients between 6 min walk test outcomes and various parameters.

Variables	Walk distance		SpO_2_ at the end of 6MWT		Borg scale at the end of 6MWT	
	*r*	*p*	*r*	*p*	*r*	*p*
Age	−0.20	0.19	−0.27	0.07	0.06	0.68
Height	0.23	0.12	0.16	0.29	−0.10	0.51
Weight	0.31	0.04	−0.10	0.51	−0.03	0.84
BMI	0.26	0.08	−0.27	0.07	0.05	0.76
FVC	0.49	0.0005	0.12	0.44	−0.23	0.12
% FVC	0.39	0.007	−0.04	0.80	−0.22	0.14
DLco	0.61	<0.0001	0.47	0.001	−0.38	0.01
% DLco	0.46	0.002	0.49	0.0007	−0.42	0.004
PaO_2_	0.43	0.002	0.43	0.003	−0.30	0.04
PaCO_2_	0.17	0.27	−0.09	0.56	−0.12	0.47
Mean AP	−0.01	0.95	0.02	0.90	0.08	0.60
RAP	0.10	0.52	0.26	0.09	−0.04	0.79
Mean PAP	−0.27	0.07	−0.50	0.0003	0.32	0.03
PCWP	0.004	0.98	0.01	0.94	−0.02	0.90
CO	−0.05	0.76	0.16	0.29	0.14	0.36
Cardiac index	−0.13	0.65	0.10	0.52	0.17	0.26
PVR	−0.33	0.02	−0.63	<0.0001	0.24	0.12

The number of patients was 46 except for DLco, which numbered 44.

BMI: body mass index; FVC: forced vital capacity; % FVC: % predicted FVC; DLco: diffusing capacity of carbon monoxide; % DLco: % predicted DLco; PaO_2_: partial arterial pressure of oxygen; PaCO_2_: partial arterial pressure of carbon dioxide; AP: systemic arterial pressure; RAP: right arterial pressure; PAP: pulmonary artery pressure; PCWP: pulmonary capillary wedge pressure; CO: cardiac output; PVR: pulmonary vascular resistance; SpO_2_: transcutaneous oxygen saturation; 6MWT: 6 min walk test.

**Table 4:** Results of stepwise multiple regression analysis for walk distance and SpO_2_ and the Borg scale at end of 6 min walk test.

Variables	*r*	*p*
For walk distance		
Weight	—	—
% FVC	0.37	0.01
% DLco	0.42	0.003
PaO_2_	—	—
PVR	—	—

For SpO_2_ at end of 6MWT		
% DLco	0.31	0.001
PaO_2_	—	—
Mean PAP	—	—
PVR	−0.51	<0.0001

For the Borg scale at end of 6MWT		
% DLco	−0.42	0.004
Mean PAP	—	—

The number of patients was 44 because 2 were excluded from the analysis due to absence of % DLco. *r* is presented as a partial correlation coefficient. *r* is presented only for variables which were selected as significant by the stepwise multiple regression analysis. Cumulative *r* ^2^ was 0.35 (*p* = 0.0002) for walk distance, 0.46 (*p* < 0.0001) for SpO_2_ at end of 6MWT, and 0.18 (*p* = 0.005) for the Borg scale at end of 6MWT, respectively.

% FVC: predicted forced vital capacity; % DLco: % predicted diffusing capacity of carbon monoxide; PaO_2_: partial arterial pressure of oxygen; PAP: pulmonary artery pressure; PVR: pulmonary vascular resistance; SpO_2_: transcutaneous oxygen saturation; 6MWT: 6 min walk test.
